# *RAPID*: an *ImageJ* macro for indexing electron diffraction zone axis spot patterns of cubic materials

**DOI:** 10.1107/S1600576724010215

**Published:** 2024-11-26

**Authors:** Thomas E. Weirich

**Affiliations:** a Gemeinschaftslabor für Elektronenmikroskopie (GFE) RWTH Aachen University Ahornstrasse 55 52074Aachen Germany; SLAC National Accelerator Laboratory, Menlo Park, USA

**Keywords:** electron diffraction, zone axis spot patterns, Kikuchi patterns, transmission electron microscopy, orientation determination, cubic symmetry, ratio methods, *ImageJ* macros, *RAPID*, automated indexing

## Abstract

As an extension to previous work, the *ImageJ* macro script *RAPID* (ratio-method pattern indexing) has been developed to allow instant indexing of calibrated and uncalibrated zone axis aligned electron diffraction patterns of cubic lattices using the *R_n_* ratio principle. The program can be used to index zone axis aligned selected-area electron diffraction patterns, nanobeam electron diffraction patterns, transmission electron microscopy (TEM) Kikuchi patterns and even fast Fourier transforms of high-resolution (scanning) TEM images. The program allows the user to quickly assess whether the material under investigation belongs to the cubic crystal system, is pseudo-cubic or is not cubic at all by adjusting the boundary parameters and allowed errors for lattice indexing. The software also allows one to distinguish between the *P*, *I* and *F* Bravais lattices for certain zone axis directions. For calibrated diffraction patterns, the lattice parameters can be obtained, allowing verification of the material under investigation or phase identification in connection with a structural database. In addition, the program can be employed for determination or verification of the used instrument’s camera constant when reference materials are used. Therefore, it is a convenient tool for on-site crystallographic analysis in TEM laboratories.

## Abstract 2

*RAPID* is an *ImageJ* macro script developed for the quick determination of sample orientation and indexing of calibrated and uncalibrated zone axis aligned electron diffraction patterns from materials with a cubic crystal structure.

## 1. Introduction

Despite considerable progress in the development of novel materials for an ever-widening range of new technological applications, research in the field of metallic alloy systems, ceramics and semiconductors continues to be dominated by relatively small unit cell phases with cubic or pseudo-cubic symmetry. In the context of transmission electron microscopy (TEM), reliable information about the specimen orientation is often essential for successfully conducting experiments such as weak-beam dark-field imaging or high-resolution (scanning) TEM [HR(S)TEM] (Williams & Carter, 2009 ▸). A standard method for achieving these goals is the acquisition of zone axis aligned selected-area electron diffraction (SAED) patterns, which are readily available for on-site analysis thanks to modern pixelated cameras (MacLaren *et al.*, 2020 ▸; Nord *et al.*, 2020 ▸; Paterson *et al.*, 2020 ▸). In the next step, users must also be able to quickly index the recorded electron diffraction pattern and determine the crystal orientation to verify that the experimental conditions for the intended experiment are set up correctly. Given that the indexing procedure may need to be repeated several times during a TEM investigation, it became evident a long time ago that computer-aided methods are the best choice for this task (Goehner & Rao, 1977 ▸; Fraundorf, 1981 ▸; Prantl, 1984 ▸). A brief overview of the general strategies and computer programs that can be used for this task is provided in Section 2.

As an extension to previous work, the *ImageJ* macro script *RAPID* (ratio-method pattern indexing) has been developed to allow instant indexing of calibrated and uncalibrated zone axis aligned electron diffraction patterns of cubic lattices. *RAPID* uses the *R_n_* ratio principle that has recently been reported for the manual indexing of such patterns (Weirich, 2024*a* ▸). A discussion of the specific conditions and possibilities for indexing cubic lattices is given in Section 3, and the implementation of *RAPID* is described in Section 4. Some practical examples are given in Section 5.

## 2. Prospects and limitations of computerized electron diffraction pattern indexing

Due to the high accelerating voltages between 80 and 300 kV used in TEM, the de Broglie wavelength of the electrons is very small (0.04176–0.01969 Å for 80 and 300 kV), and thus the radius of the Ewald sphere is correspondingly large in relation to the reciprocal lattice base vectors. Ignoring its small curvature, the Ewald sphere can then be approximated as a planar section through reciprocal space. Thus, a single electron diffraction pattern recorded from a sufficiently thin crystal, with large excitation errors, can be considered as an intensity-weighted section through the 3D reciprocal lattice of the crystal. This simple picture, however, neglects any effects that may give rise to additional (forbidden by crystal symmetry) diffraction spots (Cowley & Moodie, 1959 ▸). If this section through reciprocal space shows (accidentally or intentionally) a symmetrical distribution of the diffraction spots, the crystal under investigation is usually aligned along one of its own zone axes. Such zone axis patterns can always (without any knowledge about the underlying 3D lattice) be indexed according to one of the four 2D lattices: oblique, rectangular, square and hexagonal (Hahn & Looijenga-Vosb, 2016 ▸). Computer programs that allow this basic type of indexing are *e.g. EXTRAX* (Dorcet *et al.*, 2010 ▸), *ELD* (Zou *et al.*, 1993*a* ▸,*b* ▸), *AutoSADP* (Wu *et al.*, 2012 ▸), *EDP* (Brink & Wei Tam, 1996 ▸) and *QED* (Belletti *et al.*, 2000 ▸). The subsequent step, however, is critical, since the *HK* indices of the zone axis pattern must now be re-indexed and transformed into *hkl* Laue indices of the 3D reciprocal lattice of the crystal, according to
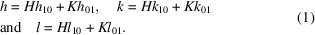
Here, the Laue indices *h*_10_*k*_10_*l*_10_ and *h*_01_*k*_01_*l*_01_ represent the 3D Laue indices that correspond to the 2D lattice defining reflections *HK* = 10 and *HK* = 01 in the zone axis pattern. This brings us to the point where it is necessary to distinguish between two cases: (*a*) the crystal lattice of the material under investigation is truly unknown or (*b*) the structure is known, and the structural parameters can be obtained from the literature or one of the reference databases for crystal structures.

For case (*a*), of truly unknown structure, it is usually not possible to derive the required *hkl* indices for the 2D lattice defining reflections from a single zone axis pattern. The only exception is the cubic lattice, which we will discuss later in more detail. In all other instances, another pattern must be obtained from at least one different direction at a known angular difference to the first (Andrews *et al.*, 1968 ▸; Lyman & Carr, 1993 ▸). This approach for reconstruction of the reciprocal lattice is known as the rotation method (Vainshtein, 1964 ▸), which is employed in several computer programs for *ab initio* unit cell determination, including *TRICE* (Zou *et al.*, 2004 ▸), *UnitCell Tools-Two Patterns* (Shi, 2022 ▸), *ProcessDiffraction* (Lábár, 2005 ▸), *QED* (Belletti *et al.*, 2000 ▸) and *PIEP* (Miehe, 2002 ▸). Other researchers have employed a similar methodology but have not made their programs accessible to the public (*e.g.* Fraundorf, 1981 ▸; Prantl, 1984 ▸; Yang *et al.*, 2017 ▸; Zaefferer, 2000 ▸). However, the rotation method has made enormous progress in recent years since its development in late 2000 (Kolb *et al.*, 2007 ▸, 2008 ▸; Zhang *et al.*, 2010 ▸). The resulting development of automated acquisition of 3D electron diffraction tomography data in (scanning) transmission electron microscopes or dedicated instruments has made this approach nowadays a routine method for structure analysis of small volumes (*e.g.* Gemmi *et al.*, 2019 ▸; Plana-Ruiz *et al.*, 2020 ▸; Samperisi *et al.*, 2022 ▸; Truong *et al.*, 2023 ▸; Simoncic *et al.*, 2023 ▸).

In case (*b*), when only a single conventional zone axis aligned electron diffraction pattern is available, a solution for the indexing problem can only be found if the structure is known and the structural parameters can be obtained from the literature or one of the reference databases. For the latter, the commonly agreed strategy is first to determine the *d* spacings and angles between the diffraction spots from a calibrated electron diffraction pattern and then to compare these with the calculated values from known structures (Andrews *et al.*, 1968 ▸; Lyman & Carr, 1993 ▸). Programs that use this approach to index single electron diffraction patterns are the phase-identification modules in *ELD* (Zou *et al.*, 1993*a* ▸,*b* ▸), *ProcessDiffraction* (Lábár, 2005 ▸), *CrysTBox* (Klinger, 2017 ▸), *PTCLab* (Gu *et al.*, 2016 ▸), *eSpot* (http://www.crystorient.com), *JEMS* (https://www.jems-swiss.ch) and the online program *Odpin* (https://www.odpin.com/). This method becomes even more powerful when linked with a structural database, where chemical composition can be used as an additional constraint to narrow down the number of possible solutions. This approach for phase identification and indexing of electron diffraction patterns is also used by the new *SAED extension* module in the latest ICDD PDF-5+ database (Kabekkodu *et al.*, 2024 ▸). Another popular approach known as 4D STEM (*e.g.* Zuo *et al.*, 2022 ▸) should also be mentioned in this context. Here, a large number of nanobeam electron diffraction (NBED; Cowley, 1999 ▸) patterns are first collected by scanning the electron probe over the sample (scanning electron nano diffraction, SEND; Zuo & Tao, 2011 ▸) without tilting the sample, and then automated crystal orientation mapping (ACOM) using templates, generated from the crystal data of known structures, is used to find the best match. This allows one not only to obtain the Laue indices for each reflection but also to generate phase distribution and orientation maps, which is the main objective of this method (Rauch *et al.*, 2010 ▸; Lábár, 2022 ▸). ACOM does not require the SEND patterns to be aligned along one of the zone axis directions, since crystal tilt is a parameter (among others) that is varied within the generation of the templates for the pattern matching.

In summary, the complete indexing of an electron diffraction pattern either requires one to reconstruct the reciprocal lattice from a tilt series with *n* ≥ 2 diffraction patterns or requires first the determination of the crystal orientation via search–match methods using known structures. As mentioned earlier, the only exception without these restrictions is the cubic crystal system, since here the *hkl* Laue indices of the 3D lattice are uniquely related to the *d* spacings in the 2D pattern:
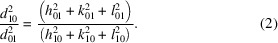
The only task then is to find the matching ratios for the *d* spacings and *hkl* ratios calculated for the cubic 3D lattice (*R*_*n*_ ratio method; *e.g.* Weirich, 2024*a* ▸). Moreover, the obtained result can be checked for reliability of the solution by comparing the calculated angle between the corresponding *hkl* planes in three dimensions with the experimental angle measured between the diffraction spots indexed with *HK* = 10 and *HK* = 01. If the agreement of the calculated and measured data is within the allowed error range, the indexing of the diffraction spots can be considered as promising (for a more detailed description of the method see Section 3). As a convenient side effect, TEM Kikuchi diffraction patterns can also be indexed by this method if they show a zone axis. In this case, the *d* spacings calculated from the width of the Kikuchi bands are used instead of those calculated from the positions of the diffraction spots. The general procedure for indexing TEM Kikuchi patterns without the need for a zone axis can be found elsewhere (*e.g.* Morawiec, 2020 ▸; Zaefferer, 2000 ▸). Available computer programs for the simulation, indexing and analysis of TEM Kikuchi diffraction patterns include *Tompas* (Xie & Zhang, 2020 ▸), *JEMS* (https://www.jems-swiss.ch) and *KLine* (http://www.crystorient.com).

## 3. The *R*_*n*_ ratio method for indexing cubic zone axis spot patterns

As outlined elsewhere in more detail (Weirich, 2024*a* ▸), the *R*_*n*_ ratio method is founded upon the relationship in equation (3), shown below, where *r*_*A*_ and *r*_*B*_ are the distances of two reflection spots in a zone axis pattern from the center, and *d* and the triplets *hkl* correspond to the interplanar distances and Laue indices, respectively:
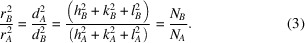
Hence, indexing of a cubic spot pattern involves firstly determining the ratio *r*_*B*_/*r*_*A*_ from the experimental pattern and then finding a match with the ratio (*N*_*B*_)^1/2^/(*N*_*A*_)^1/2^, which provides a trial set of Laue indices for the two reflections. The corresponding vector of the zone axis [*uvw*], which is equal to the direction of the electron beam, is perpendicular to the plane defined by the two (non-opposite or in-line) reciprocal space vectors of reflection spots *A* and *B*, and can be calculated by the cross product according to
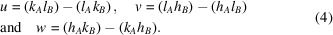
A first proof that the determined *hkl* indices represent a possible solution is made by verifying that the experimental determined interplanar angle ϕ_*A*−*B*_ between the two reflection spots agrees with calculation. This is carried out using the relation
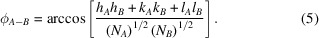
To be sure that the found match truly belongs to a cubic lattice, the ratio and angle calculation must be repeated for a third diffraction spot *C*. Therefore, the minimum required information from the experimental data for indexing a zone axis pattern of a cubic lattice are the measured distances *r*_*A*_, *r*_*B*_ and *r*_*C*_ and the angles ϕ_*A*−*B*_ and ϕ_*A*−*C*_. The selected distances must correlate with the three shortest reciprocal space vectors observed in the diffraction pattern for the approach to function correctly. For the here used indexing scheme, the Laue indices of spot *C* are calculated according to *h*_*A*_*k*_*A*_*l*_*A*_ − *h*_*B*_*k*_*B*_*l*_*B*_ = *h*_*C*_*k*_*C*_*l*_*C*_ from the indices of spots *A* and *B* [see also Weirich (2024*a* ▸,*b* ▸)]. If the camera constant CC – the magnification factor of the reciprocal lattice in the diffraction experiment – is known, the measured distances *r* (in pixel units) also yield directly the interplanar *d* spacings from equation (6) and the lattice parameter *a* from equation (7):
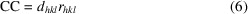
and
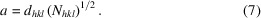


## 4. Implementation of the *R*_*n*_ ratio method as an *ImageJ* macro program

**Figure 1:**
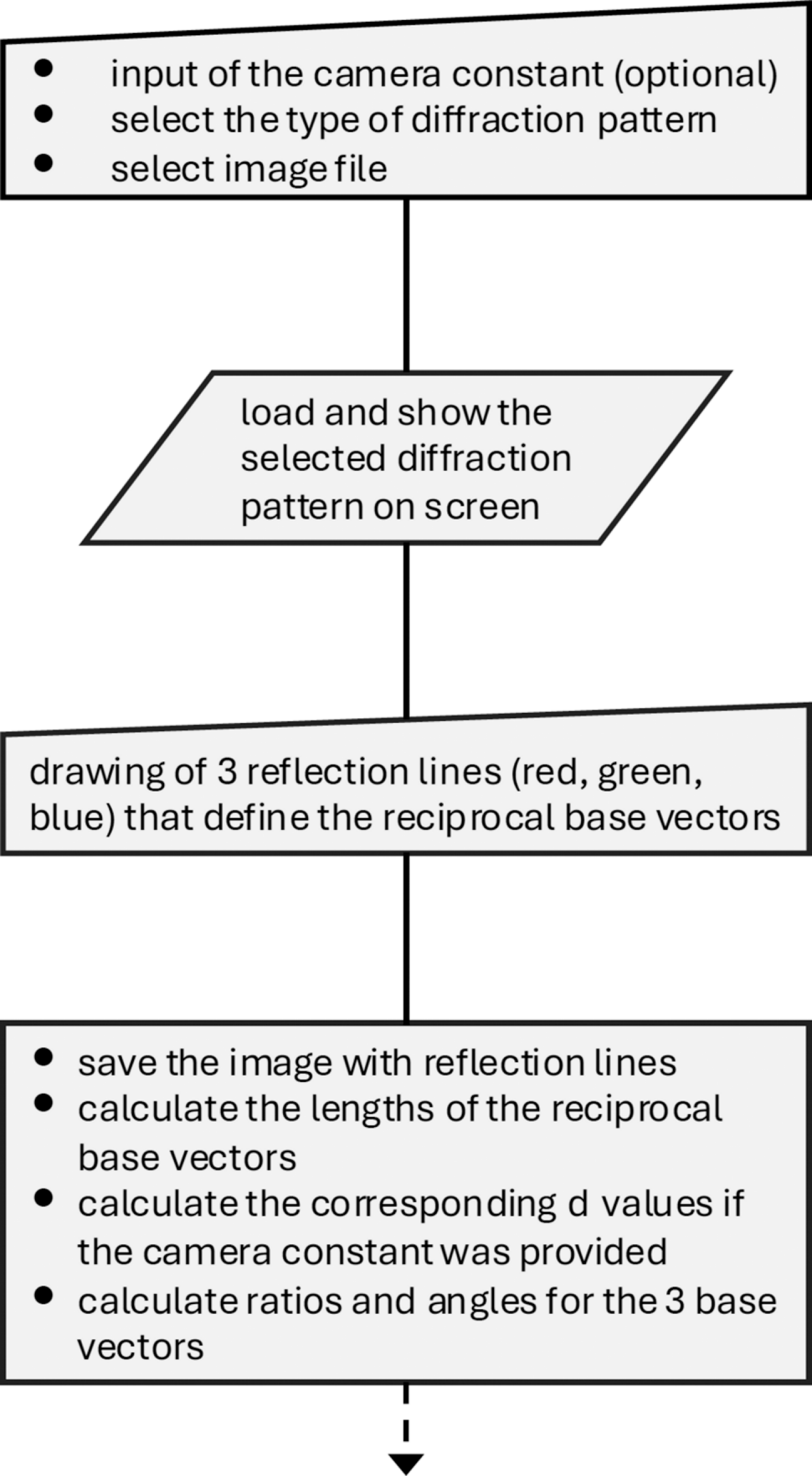
Flowchart part I of the *ImageJ* macro program *RAPID*.

The macro code of *RAPID* was developed using the current freely available *FIJI* distribution of *ImageJ* (Schindelin *et al.*, 2012 ▸; Schneider *et al.*, 2012 ▸). The macro code is fully accessible and thus can be easily adjusted and extended to meet the specific needs of the user [see, for an introduction, Ferreira & Ehrenfeuchter (2022 ▸)]. The flowchart of the macro, which illustrates the individual steps up to the determination of the zone axis, is shown in Figs. 1 ▸ and 2 ▸. The corresponding flowchart of the user interface is shown in Fig. 3 ▸. The macro can be executed by loading the code file from the program editor or via the ‘Plugins’ menu. In addition, the macro can be permanently added to the list of available tools, making it easily accessible for frequent use.

The initial stage of processing a diffraction pattern with *RAPID* is the request to the user to provide the camera constant (optional), to indicate the type of diffraction pattern (SAED or Kikuchi pattern) and to choose an image file that is compatible with *ImageJ* (Fig. 3 ▸, No. 1). Note that a DM3 reader plugin for *ImageJ* is also available (https://imagej.net/ij/plugins/DM3_Reader.html), which enables images to be read in the *DigitalMicrograph* format (Gatan Inc., Pleasanton, CA, USA) without the necessity of converting the images into one of the common bitmap image formats prior processing. In the following step, the selected image is opened and displayed, and the user is asked to draw lines between opposite diffraction spots *hkl* and *hkl* using *ImageJ*’s built-in *Straight Line* tool for defining the three shortest reciprocal base vectors (Fig. 3 ▸, No. 2). The rule here is to start with the two opposite diffraction spots that are closest to the center (red line), then continue with the next closest pair of spots (green line) and finish by drawing a line between the third shortest pair of diffraction spots (blue line). For all type II patterns, and a few others, the second- and third-shortest reciprocal space vectors are essentially the same length (Weirich, 2024*a* ▸). In such cases, there is no priority among these two, but the shortest vector (the red line) must be defined before them. In the special case of a 〈111〉 pattern, where all three shortest vectors have the same length, there exists no such priority rule (see the example in Fig. 6). To improve the accuracy of the measurement, the macro code can also handle lines between higher-order reflections as long as the number of reflections in each line is equal to 2*n*, where *n* can be an integer between 1 and 4 in the current version of *RAPID* (Fig. 3 ▸, No. 3). When a zone axis aligned Kikuchi pattern is in use, this function is not available, since only the width of the Kikuchi band (equal to *n* = 0.5) will be used. After measurement and calculation of the user-defined three shortest reciprocal space vectors, the image with the lines is automatically saved on the hard drive for later documentation. If the camera constant CC (in ångstrom pixel units) has been provided, the corresponding *d* values are calculated and given in the report. If CC is initially set to zero or a negative value, the program will interpret this as ‘unknown’ and all further calculations and reporting will be based on the length in pixel units. All the processing described so far is also shown in part I of the flowchart in Fig. 1 ▸.

**Figure 2:**
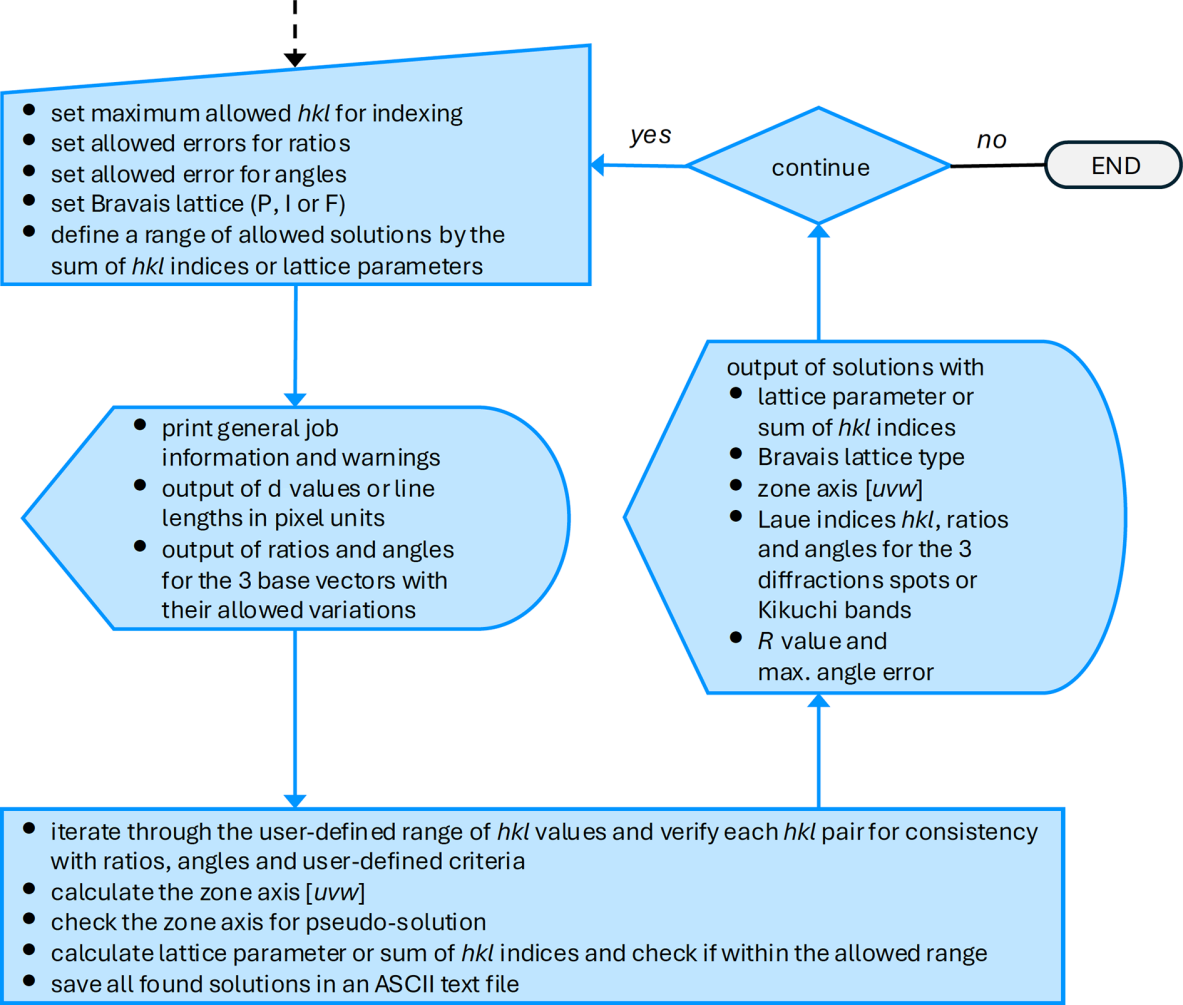
Flowchart part II of *RAPID*.

**Figure 3:**
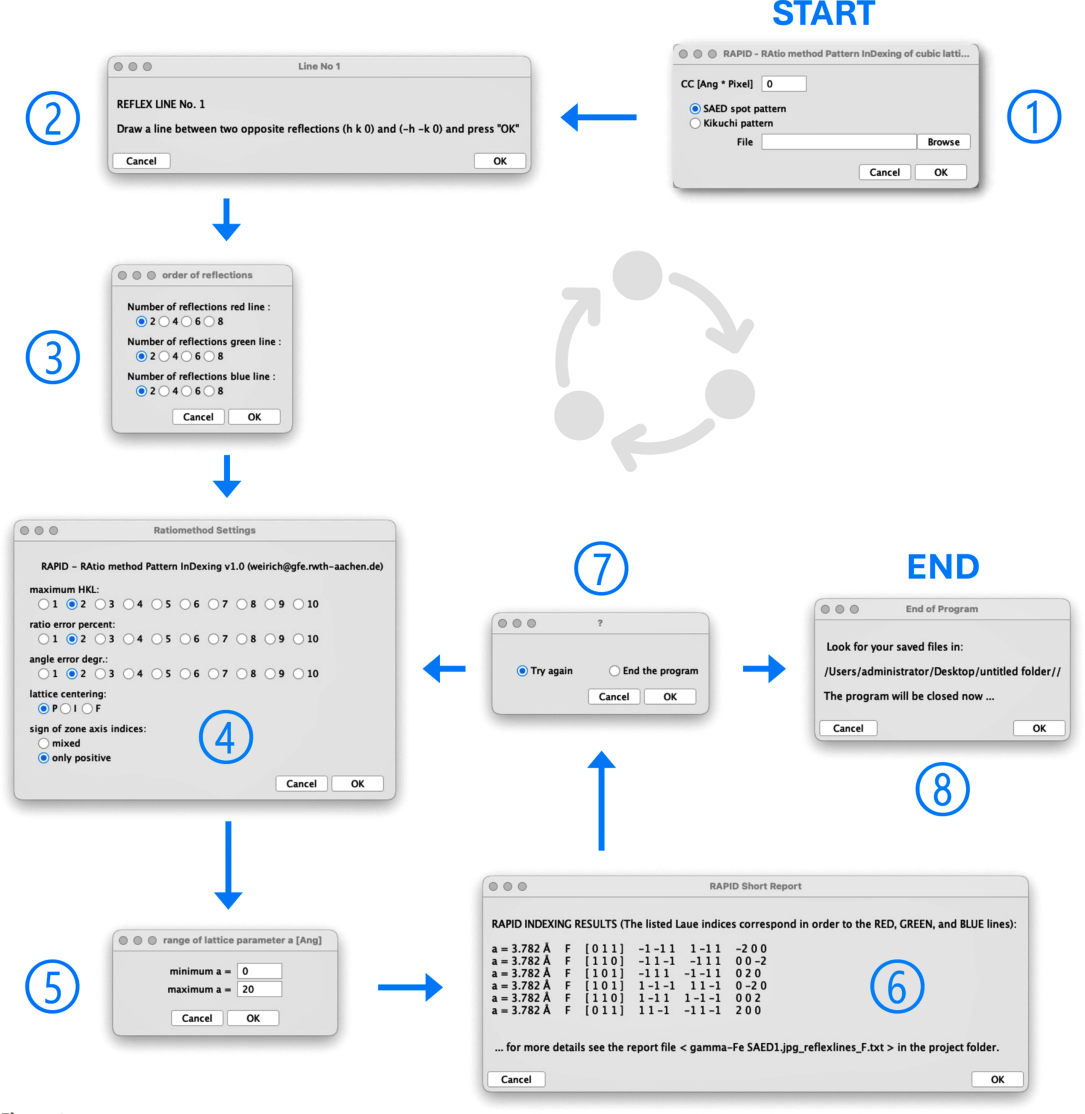
Flowchart of the user interface of *RAPID*.

All subsequent program descriptions refer to part II of the flowchart, shown in Fig. 2 ▸. The program routines in this part, such as user input of the indexing parameters, calculations and output of results, are arranged within an (infinite) while loop, which can be interrupted by the user after each cycle to stop execution of the program (Fig. 3 ▸, No. 7 and 8). At the first stage within the while loop, the user is asked to set some parameters that affect the indexing of the measured data (Fig. 3 ▸, No. 4). These parameters are the range of maximum allowed *hkl*, permitted errors for ratios and angles, and the type of Bravais lattice considered. Then a range of allowed solutions can be defined either by the sum of the *hkl* indices of the shortest and second-shortest lattice vectors or by a certain range of lattice parameters, *e.g.* report all solutions with lattice parameter *a* between 3.5 and 4.5 Å (Fig. 3 ▸, No. 5). However, this functionality to limit the number of solutions by lattice parameters is only available if the camera constant CC is not set to zero (or a negative value). The provided lattice parameter is only a rough estimate and not a high-precision value, as the spot positions are defined by a mouse-click on the computer screen and not by a sophisticated algorithm looking for the exact peak centers. Nevertheless, the lattice parameter provided is quite useful since it can help to eliminate false solutions from the results listing, or for a quick check on the material or the camera constant. During the further processing, the macro provides a report on some general information about the running job, such as the name of the image file, the type of diffraction pattern in use and whether the camera constant has been provided by the user. There are also some warnings that need to be considered when interpreting the results, such as the user-defined error ranges or limits for the *hkl* indices. If the camera constant has been defined, the *d* values of the three base reflections will be printed; otherwise their lengths will be given in pixel units. Moreover, the calculated ratios for *r*_*B*_/*r*_*A*_ and *r*_*C*_/*r*_*A*_, and the experimental angles ϕ_*A*−*B*_ and ϕ_*A*−*C*_ between them, will be listed in the output. Within the core indexing routine, the macro iterates through the user-defined range of *hkl* values and calculates trial ratios and angles using equations (3) and (5). Each *hkl* triple is tested for agreement with the experimental ratios and angles within the prior defined error limits. Having passed this initial check, the corresponding zone axis [*uvw*] is calculated from equation (4) and validated to avoid pseudo-solutions. A pseudo-solution in this context is, for example, the zone axis [011] for the cubic *F* lattice with ratios 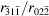 and 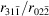 and angles 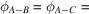

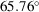
 that matches exactly the true solution [114] with ratios 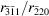 and 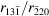 and the same angles [see Weirich (2024*b*), pp. 35 and 46]. After passing this check, an averaged lattice parameter is calculated from equation (7) or, if the camera constant has not been provided, the sum of the *hkl* indices of the shortest and second-shortest lattice vectors is calculated and evaluated to see if it falls within the allowed range. If true, the macro writes, for each valid solution, the lattice parameter (or *hkl* sum), the selected Bravais lattice type, the identified [*uvw*] zone axis, and the *hkl* Laue indices of the indexed reflections and their calculated ratios and angles to the output window. A corresponding shortened report is also shown in a separate window (Fig. 3 ▸, No. 6). Furthermore, the program provides a reliability factor for each solution according to equation (8), as well as the maximum difference between the experimental and calculated angles, as defined by equation (9):
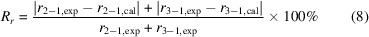
and
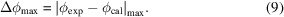
Here, *r*_2−1_ is the ratio calculated by dividing the length of the second (green) line by the length of the first (red) line. Correspondingly, *r*_3−1_ is obtained by dividing the length of the third (blue) line by the length of the first (red) line. Note that the ratios of the lengths of the lines are the same as the inverse of the *d* value ratios, *i.e.r*_2−1_ = *d*_red_/*d*_green_ and *r*_3−1_ = *d*_red_/*d*_blue_. At the end of each indexing cycle, the full screen log is automatically updated and stored for documentation purposes or for immediate inspection by the user.

## 5. Examples

### 5.1. SAED pattern: austenitic chrome–nickel–molybdenum steel

**Figure 4:**
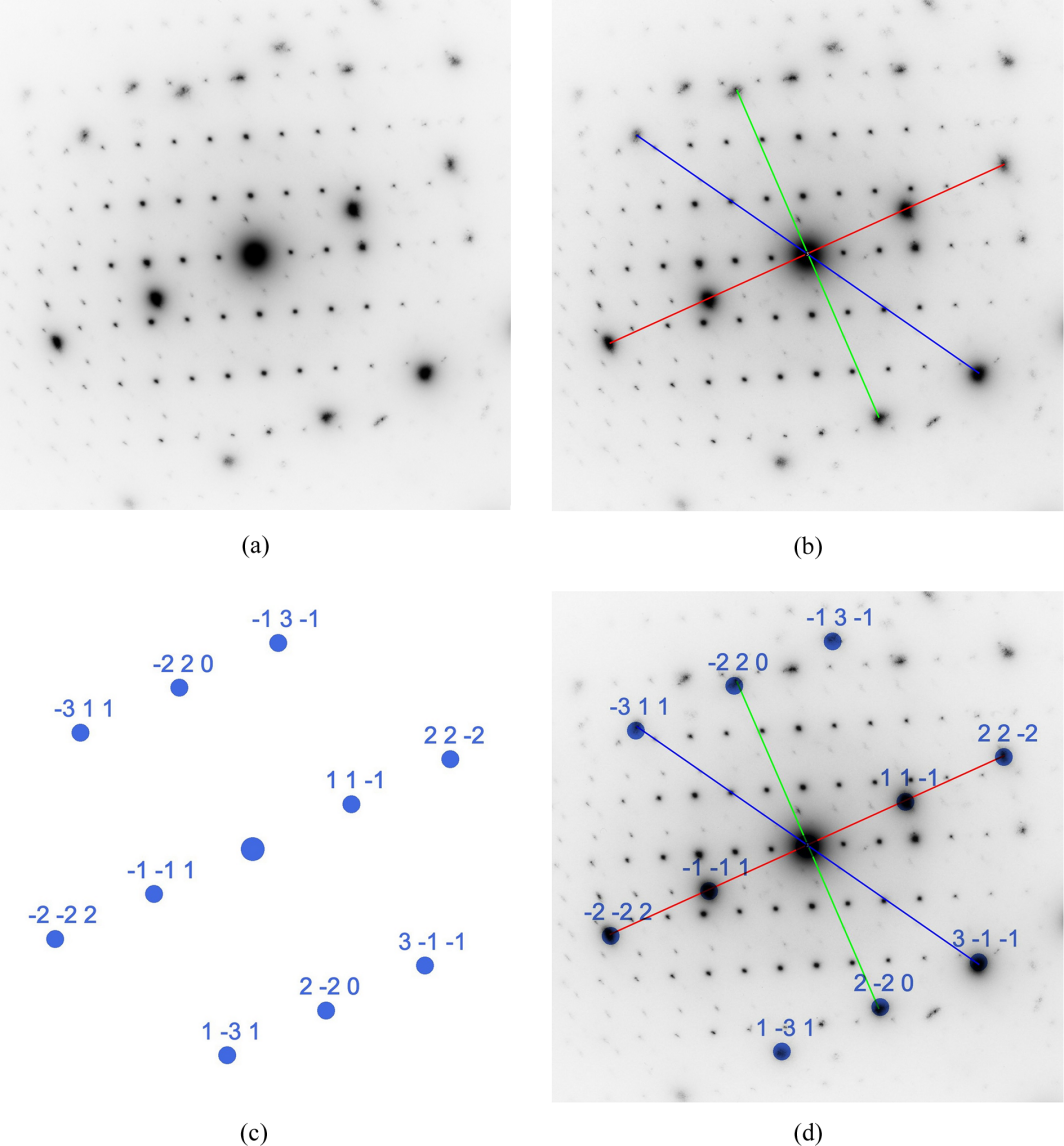
Different stages of the processing of an SAED pattern from austenitic chrome–nickel–molybdenum steel with the program *RAPID* for determining the matrix orientation. The calculated scaled spot pattern in Fig. 4 ▸(*c*) corresponds to a cubic *F* lattice with *a* = 3.704 Å and [112] orientation (see Section 5.1).

The SAED patterns used in this and the following example were obtained from a focused ion beam cross section of an austenitic chrome–nickel–molybdenum steel, which was investigated at 200 kV in a FEI Tecnai F20 transmission electron microscope at the author’s laboratory. The diffraction pattern in Fig. 4 ▸ has been chosen as an example since it shows both a zone axis aligned steel matrix grain and a zone axis aligned M_23_C_6_ carbide. The following section will demonstrate how *RAPID* can be employed to determine the zone axis of the austenitic matrix grain. In this case, the camera constant (CC = 590 Å pixel) was provided to the program to verify the austenitic matrix by its lattice parameter. As shown in Fig. 4 ▸(*b*), the shortest reciprocal space vector (red) was defined by a line that covers four reflections, whereas the second-shortest vector (green) and the third-shortest vector (blue) were defined by lines that cover only two reflections each. These settings have been made in the menu at step No. 3 in Fig. 3 ▸. In the user menu at stage No. 4, the default settings for maximum *hkl* (default = 2), allowed ratio error (default = 3%) and angle error (default = 2°) were all kept. Finally, a filter for only positive *uvw* values was set. The lattice parameter limit was also left at the default range between 0 and 20 Å (stage No. 5 in Fig. 3 ▸). Evaluation of the measured lines yields *d*_red_ = 2.149 Å, *d*_green_ = 1.304 Å and *d*_blue_ = 1.116 Å, with the ratios *d*_red_/*d*_green_ = 1.648 and *d*_red_/*d*_blue_ = 1.926. The determined angle between the diffraction spots on the red and green lines is 89.03° and between the spots on the red and blue lines is 59.25°. With these data and settings, *RAPID* found matches only for a cubic *F* cell with 〈112〉 orientation and lattice parameter *a* = 3.704 Å (see Table S1 of the supporting information for the index log). As this zone axis is unambiguous for the *F* lattice [see Table 1 of Weirich (2024*a* ▸)], no solutions were obtained when testing for the *P* and *I* lattices. For the solution with the specific zone axis [112], the determined Laue indices are 111 (red), 220 (green) and 311 (blue). The *RAPID* calculated (ideal) ratios are *d*_red_/*d*_green_ = 1.633 and *d*_red_/*d*_blue_ = 1.915. The ideal in-plane angles between the reciprocal space vectors for this zone axis between the red and the green vector are 90.0° and between the red and the blue vector are 58.52° [for numerical and graphical verification, see Weirich (2024*b* ▸), p. 37]. A theoretical zone axis pattern for the [112] orientation was calculated from the determined Laue indices and scaled by the camera constant [Fig. 4 ▸(*c*)]. Superimposing the calculated pattern onto the experimental pattern with the indicated reflection lines [Fig. 4 ▸(*d*)] shows a good match that confirms the correctness of the found zone axis orientation for the austenitic matrix.

### 5.2. SAED pattern: M_23_C_6_ precipitate in austenitic chrome–nickel–molybdenum steel

**Figure 5:**
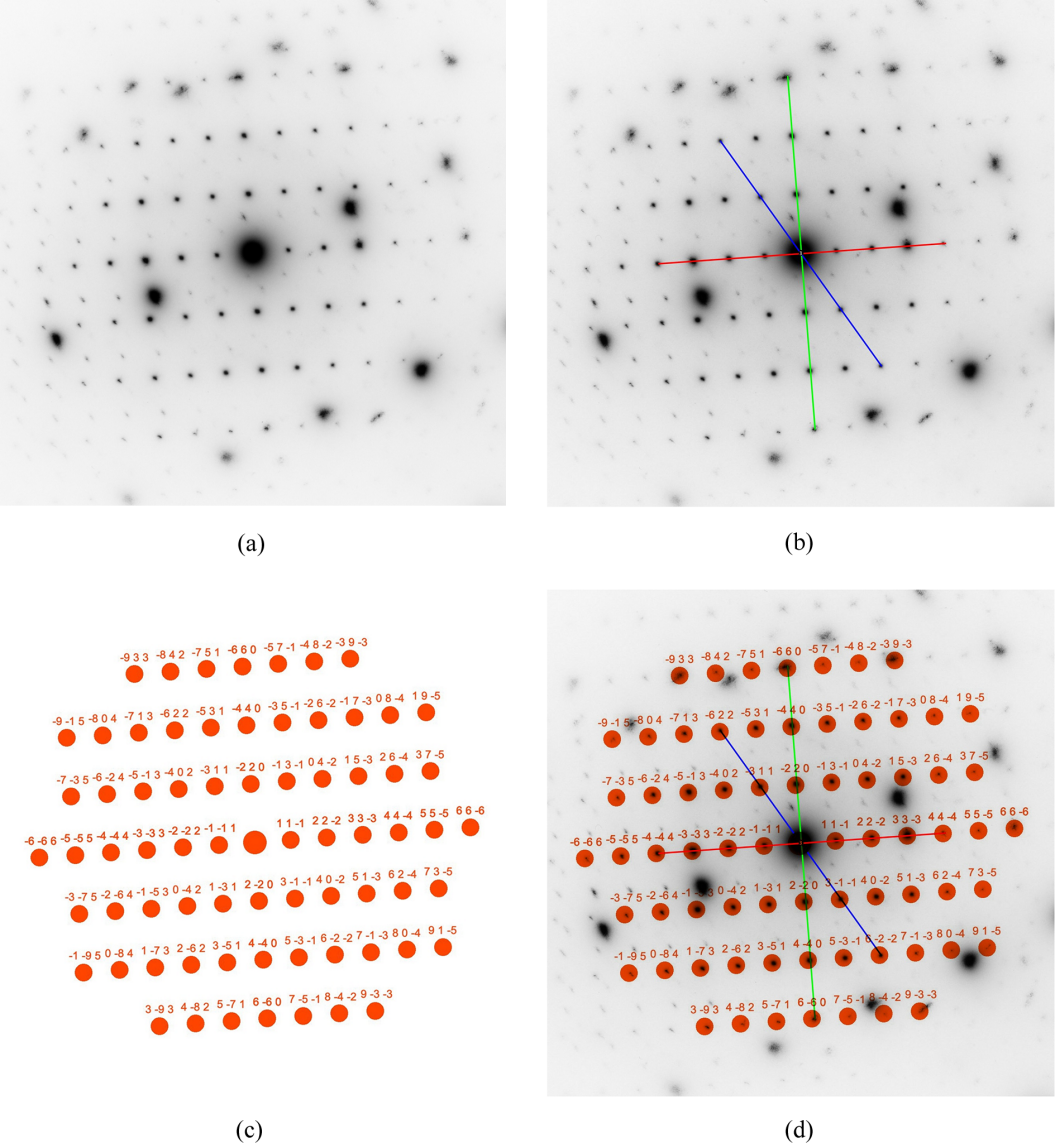
Different stages of the processing of an SAED pattern from austenitic chrome–nickel–molybdenum steel with the program *RAPID* for determining the orientation of an M_23_C_6_ precipitate. The calculated scaled spot pattern in Fig. 5 ▸(*c*) corresponds to a cubic *F* lattice with *a* = 11.069 Å and [112] orientation (see Section 5.2)

This section demonstrates how *RAPID* can assist in identifying a precipitate from the secondary phase reflections in Fig. 4 ▸. According to a standardless energy dispersive X-ray (EDX) analysis, the element ratio of the precipitate is about Cr:Mo:Co = 24:5:1, with a significant amount of carbon (not included in the quantification). As shown in Fig. 5 ▸(*b*), the three shortest reciprocal space vectors were determined from reflection lines covering 8 (red), 6 (green) and 4 (blue) diffraction spots. All other settings were left at their default values, as in the previous example. Since the secondary phase precipitate was assumed to be unknown, tests were carried out for the *P*, *I* and *F* lattices to check whether it was truly cubic and, if so, to which Bravais lattice type it belonged. Positive hits were only obtained from the *F*-lattice test, while the *P*- and *I*-lattice tests gave no results. The estimated lattice parameter for the identified *F* lattice was *a* = 11.069 Å, and all found zone axes are variants of the unambiguous 〈112〉 orientation, which is the same as for the austenitic matrix. For documentation, the complete log of this successful run of *RAPID* is listed in Table S2. Again, a theoretical zone axis pattern was calculated from the obtained result [Fig. 5 ▸(*c*)] and superimposed onto the experimental pattern [Fig. 5 ▸(*d*)] for proof. The true nature of the precipitate remains unclear at this stage as only the crystal system, the Bravais lattice type and an approximate lattice parameter are known. Finally, a database search was carried out, with the aim of narrowing down the phase of the precipitate. The ICDD PDF-5+ database (Kabekkodu *et al.*, 2024 ▸) matches the *RAPID*-indicated *F*-centered unit cell and the EDX-determined chemical composition best for Cr_18.4_Mo_4.6_C_6_ (PDF file No. 01-082-5716) with *a* = 10.9 Å. The same match was found in the ICDD database (Zagorac *et al.*, 2019 ▸; collection code 617519), so it is legitimate to assume that the precipitate has a D8_4_(M_23_C_6_) type of structure.

### 5.3. HRTEM image: orientation of an ultrasmall superparamagnetic iron oxide nanoparticle

**Figure 6:**
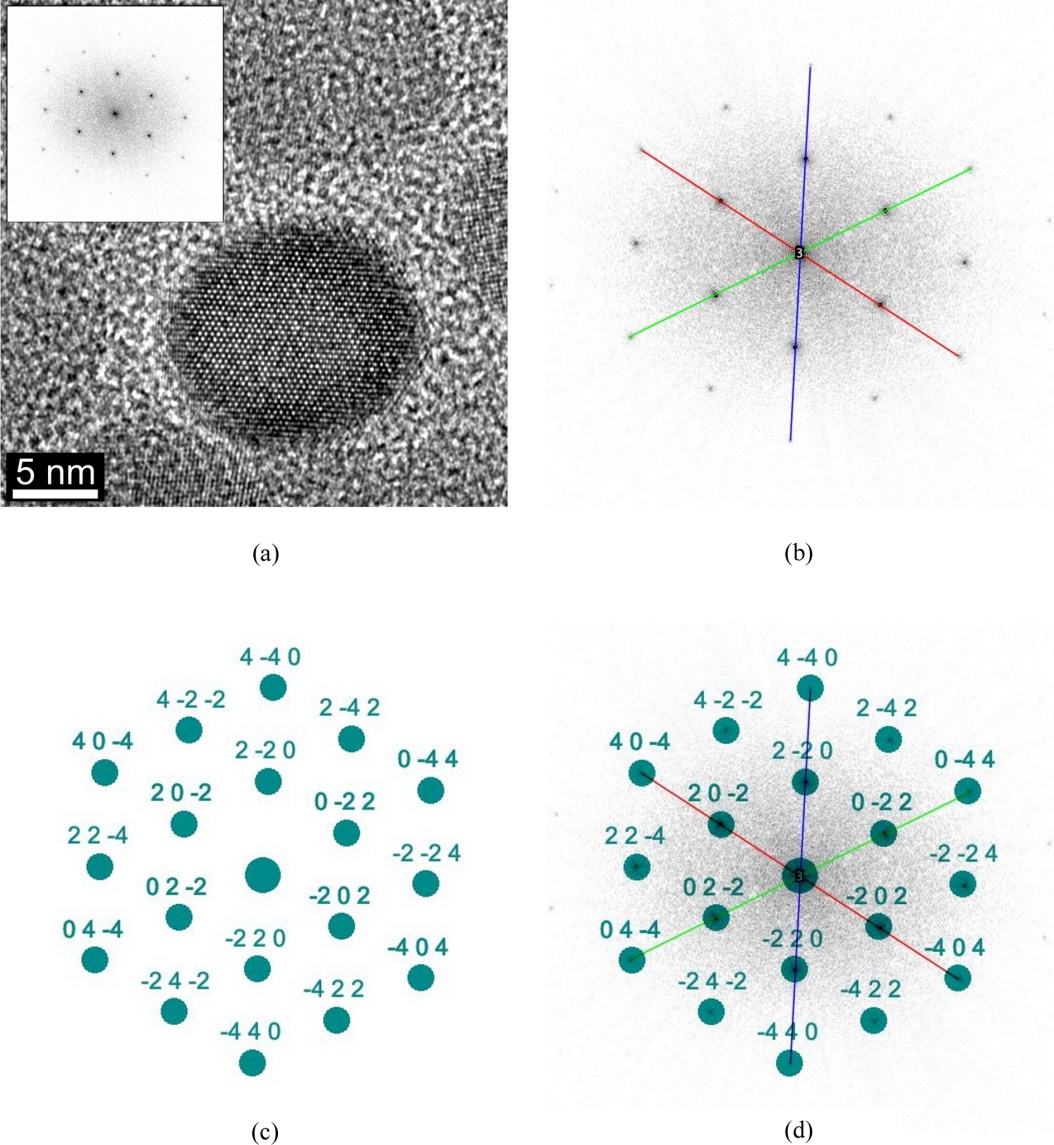
Different stages of the processing of the fast Fourier transform of an Fe_3_O_4_ nanoparticle with the program *RAPID* for determining the orientation. The calculated scaled spot pattern in Fig. 6 ▸(*c*) corresponds to a cubic *F* lattice with *a* = 8.363 Å and [111] orientation (see Section 5.3).

The following example shows a rarer use of *RAPID*, namely its application to the Fourier transform of an HRTEM image of a nanoparticle whose orientation has to be determined. The HRTEM image of the Fe_3_O_4_ nanoparticle shown in Fig. 6 ▸(*a*) was obtained during material characterization carried out as part of a study by Wang *et al.* (2011 ▸) in a FEI Tecnai F20 transmission electron microscope at the author’s facility. Prior to processing with *RAPID*, a Fourier transform of the HRTEM image was calculated using the built-in *ImageJ* function and, after calibration, processed similarly to a normal SAED pattern. Like the previous examples, three colored lines have been used to indicate the base vectors, each of which covers four reflections [Fig. 6 ▸(*b*)]. As mentioned in Section 4, in this case, where all three shortest vectors have the same length, it was not necessary to define any of the reflections first. Again, the default settings were used as in the previous examples and all three Bravais lattices were tested. The orientation determined was the 〈111〉 zone axis in all three cases, but with different calculated lattice parameters according to the different indexing for the different Bravais lattices [see Table 2 of Weirich (2024*a* ▸)]. While the results for the *P* and *I* unit cells showed *a* = 4.181 Å, the *F* cell was twice as large with *a* = 8.363 Å (Tables S3, S4 and S5). Although all three solutions agree that this is a 〈111〉 orientation, the result remains ambiguous about the true size of the unit cell. However, this is not an issue of the indexing algorithm used here but lies in this particular crystal orientation {see [111] in Table 1 of Weirich (2024*a* ▸)}. Finally, the theoretical zone axis pattern for the *F* lattice was calculated [Fig. 6 ▸(*c*)] and superimposed onto the experimental pattern of the Fourier transform [Fig. 6 ▸(*d*)]; their agreement proves that the *F* lattice is the correct solution.

### 5.4. Kikuchi pattern: matrix orientation of alloy AlZn5Mg

**Figure 7:**
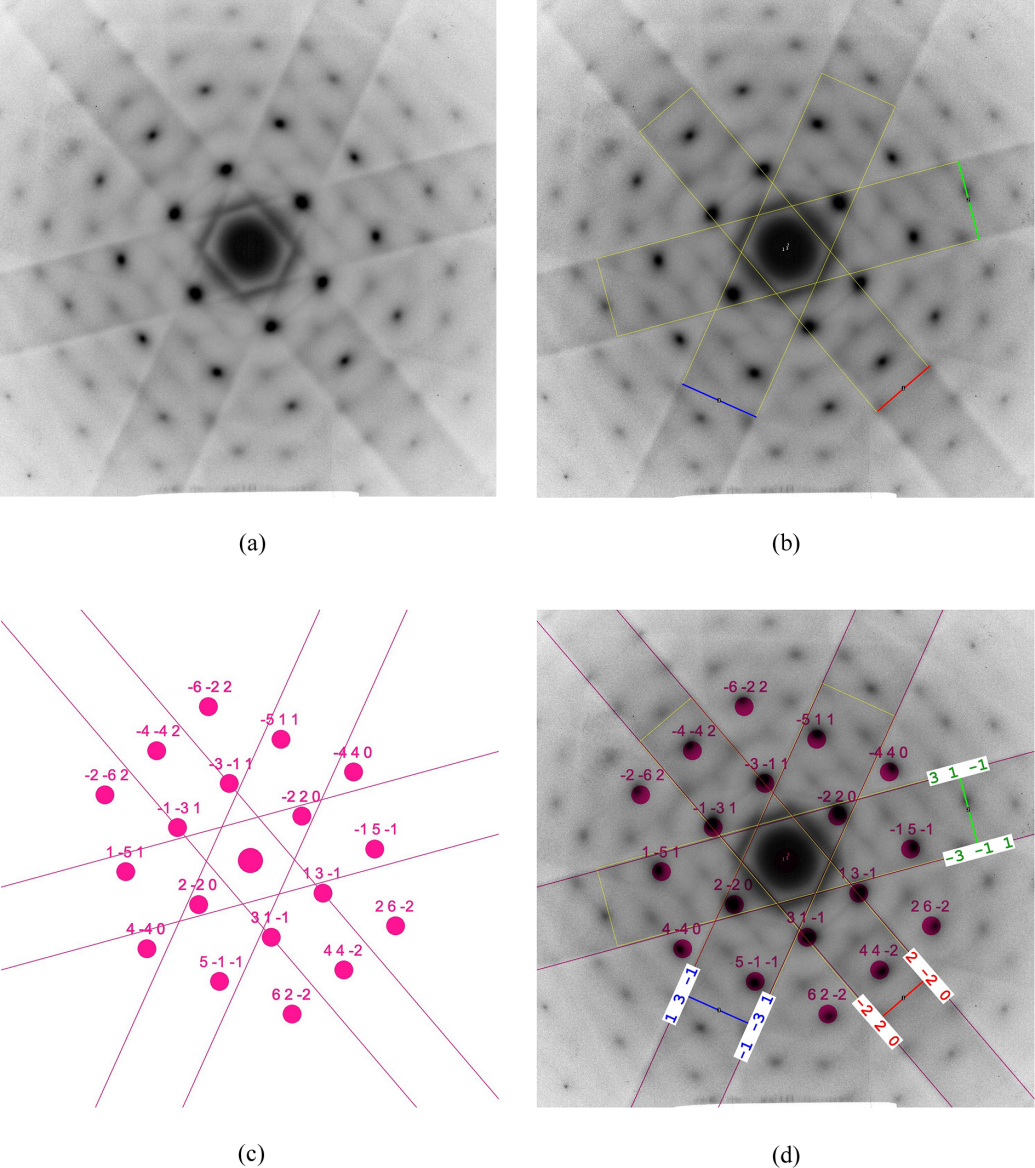
Stages of the processing of an uncalibrated SAED Kikuchi pattern from an aluminium alloy with the program *RAPID* for determining the orientation. The calculated scaled spot pattern in Fig. 7 ▸(*c*) corresponds to a cubic *F* lattice and [114] orientation (see Section 5.4).

This example demonstrates that uncalibrated images and Kikuchi diffraction patterns can also be reliably indexed by the developed code. The SAED pattern of the aluminium alloy AlZn5Mg in Fig. 7 ▸(*a*) was also used in the previous publication (Weirich, 2024*a* ▸) for the indexing of the diffraction spots, so the focus of the evaluation is now only on the Kikuchi bands. As outlined in Section 4, the difference in the processing of Kikuchi patterns compared with SAED is that the width of the Kikuchi bands is used instead of the distance between opposing pairs of diffraction spots. This must be specified in the program by selecting the correct pattern type in the first menu (Fig. 3 ▸, stage No. 1). The lines across the Kikuchi bands can be drawn anywhere as long as they are in the correct order: first the band with the smallest width, and so on. As a hint for obtaining the shortest length for each band, it may be helpful to mark the band edges with *ImageJ*’s built-in *Rotated Rectangle* tool prior to analysis with *RAPID* [see yellow frames in Fig. 7 ▸(*b*)]. Using the default parameters for indexing, as in the previous examples, initially produced no result. However, when the value of ‘maximum *hkl*’ was increased to 3, matching zone axes were readily found. Testings carried out with 2% error limits for the ratios and angles yielded only the 〈359〉 zone axis variants for the *P* and *I* lattices. The corresponding result for the *F* lattice was 〈114〉. However, a decision on the correct solution could be made by comparing the *R*_*r*_ values [equation (8)] and maximum angular errors [equation (9)] provided in the log files (Tables S6, S7 and S8). While the *R*_*r*_ values for the ratios are ∼1.2% and ϕ_max_ = 1.1° for both the *P* and *I* lattices, the residual for the *F* lattice is much lower with *R*_*r*_ = 0.56% and shows a smaller ϕ_max_ = 0.9°. This indicates that the latter is the correct solution due to the better fit with the experimental data. If the error limits for the ratios and angles are set at 1% (Fig. 3 ▸, step No. 4), only 〈114〉 is obtained for the *F* lattice, and no result is obtained for the *P* and *I* lattices. The former was therefore assumed as the correct solution in agreement with the earlier obtained result from analysis of the corresponding spot pattern (Weirich, 2024*a* ▸). The calculated diffraction pattern for zone axis [114] and its overlay on the experimental diffraction pattern are shown in Figs. 7 ▸(*c*) and 7 ▸(*d*), respectively. The indices for the analyzed Kikuchi bands are shown in Fig. 7 ▸(*d*).

### 5.5. Off-axis NBED pattern: matrix orientation of a nickel-based alloy

**Figure 8:**
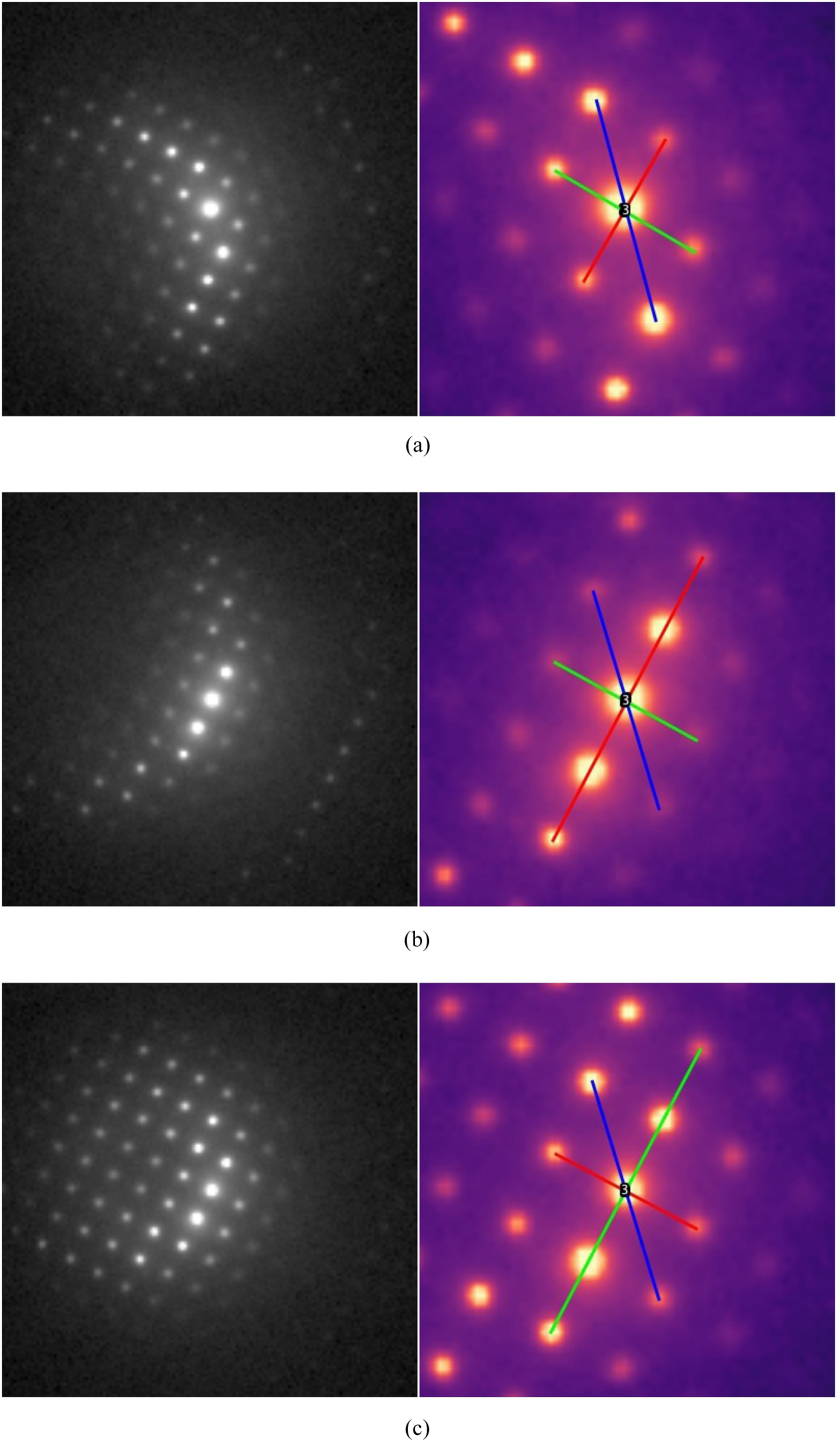
NBED patterns from the γ matrix of a nickel-based alloy containing ∼30% chromium obtained by SEND with a precession angle of 0.5° (see Section 5.5). For each pattern, the employed reflection lines for orientation determination with *RAPID* are shown in the zoomed view on the right. Despite the evident off-axis orientation of the matrix with the electron beam, the 〈001〉 zone axis was identified as the orientation for all patterns using *RAPID*. A detailed examination of the NBED patterns using the ASTAR system software revealed that the investigated crystal areas are tilted by (*a*) ∼2.6°, (*b*) 2.3° and (*c*) 1.7° from the 〈001〉 zone axis.

This example examines the potential of the *R*_*n*_ ratio approach for identifying the most closely aligned zone axis orientation from slightly off-zone-axis NBED patterns obtained by SEND data collection (Fig. 8 ▸). The NBED patterns used in this study are from the γ matrix of a nickel-based alloy containing ∼30% chromium and provide a lateral resolution between 5 and 10 nm, at least one order of magnitude below that of SAED in a non-*C*_s_-corrected transmission electron microscope. They were acquired with an ASTAR system (NanoMEGAS SPRL, Brussels, Belgium) with beam precession attached to a 200 kV JEOL JEM F200 transmission electron microscope at the author’s facility. The NBED patterns were exported from a larger block file with the NanoMEGAS ACOM software, magnified and recalibrated for analysis with *RAPID*. The processing of the NBED patterns followed the scheme described earlier. All NBED patterns shown in Fig. 8 ▸ were processed with the default values for maximum *hkl* (2) and errors for ratios and angles (2% and ±2°). For each of the patterns with the tested *P*, *I* and *F* Bravais lattices, only the 〈001〉 zone axis was identified as an orientation. However, the solutions for the Bravais lattices differed in the determined unit cell size according to *a*_*I*_ = 2^1/2^*a*_*P*_ for the *I* lattice and *a*_*F*_ = 2*a*_*P*_ for the tested *F* lattice. The latter unit cell is the correct solution for the γ phase of the nickel-based alloy. Similar to the example in Section 5.3, the determined 〈001〉 zone axis orientations cannot be uniquely deduced for one of the Bravais lattices from the ratios [see Table 1 of Weirich (2024*a* ▸)], nor can they be distinguished by the *R*_*r*_ value [equation (8)] or the maximum angle error Δϕ_max_ [equation (9)], since these descriptors are necessarily identical for all Bravais lattices. However, for calibrated patterns, the correct solution can be easily established by using the calculated lattice parameters, since these change according to the different indexing for the Bravais lattices [see Table 2 of Weirich (2024*a* ▸)]. Nevertheless, despite the evident off-axis orientation of the matrix with the electron beam, the 〈001〉 zone axis was identified as the orientation for all patterns using *RAPID*. A detailed examination of the NBED patterns using the ASTAR system software revealed that the corresponding crystalline regions of the sample are tilted by ∼2.6° [Fig. 8 ▸(*a*)], 2.3° [Fig. 8 ▸(*b*)] and 1.7° [Fig. 8 ▸(*c*)] from the 〈001〉 zone axis. This shows that, for slightly misaligned crystals, the *R*_*n*_ ratio approach used is able to correctly identify the zone axis orientation from the diffraction spots on the Laue circle of the zero zone, but cannot provide the true orientation as determined by ACOM template matching. Therefore, the *R*_*n*_ ratio method will only give correct results for the true orientation for spot diffraction patterns that are aligned ∼±0.5° or less from a zone axis, making it very limited for the evaluation of 4D STEM data. If the goal is only to determine the nearest zone axis, the ASTAR system coupled with a precession system [for a review on the method, see Midgley & Eggeman (2015 ▸)] can be used to increase the hollow cone angle beyond the current 0.5°, allowing more of the high-angle spots to be sampled and providing a more symmetric intensity distribution. However, this is a trade-off at the expense of the lateral resolution of the NBED pattern.

## 6. Discussion and conclusions

As an extension to previous work (Weirich, 2024*a* ▸), the *ImageJ* macro script *RAPID* has been developed, which facilitates the indexing of zone axis aligned electron diffraction patterns of cubic lattices by exploiting the *R*_*n*_ ratio principle without limitations and the need for pre-calculated tables. The examples shown here demonstrate several advantages of the developed program for the electron microscopy community:

(*a*) The program is simple to use (basically just drawing three reflection lines) and highly reliable for determining the correct orientation of calibrated and non-calibrated zone axis aligned SAED patterns. Moreover, the program can be employed for the indexing of zone axis aligned Kikuchi patterns and fast Fourier transforms of HR(S)TEM images in a comparable manner.

(*b*) The program enables the rapid assessment of whether the material under investigation belongs to the cubic crystal system or might be pseudo-cubic by varying the boundary parameters and permitted deviations for lattice indexing. Moreover, the program can be used by trial and error for checking the type of Bravais lattice.

(*c*) For calibrated patterns, an approximated lattice parameter is provided, which allows for verification of the material under investigation. This feature can also be employed for phase identification through a database search using the determined lattice parameter when the material is confirmed to be cubic (see examples in Sections 5.2 and 5.5).

(*d*) The program also offers a convenient method for determining (or checking) the camera constant from single-crystal diffraction patterns of known materials, since *hkl* indices and reciprocal vector lengths in pixel units of the three base reflections are provided. The camera constant can then be calculated from equation (6) using the known *d* spacings of the reference material. The latter can be obtained from freely available look-up tables, such as those published by Swanson & Tatge (1953 ▸) or in subsequent updates at NIST.

While the implementation of the *R_n_* ratio method for indexing zone axis orientated electron diffraction patterns has been successful, the program presented here is not a general solution for the analysis of diffraction patterns of non-cubic materials and/or non-orientated diffraction patterns. These cases are not covered by the method used here or may not be compatible with the current implementation, which entirely relies on the availability of three pairs of *hkl* and *h**k**l* diffraction spots as outlined in the program description. If this is a concern, consideration should be given to using alternative methods such as ACOM, which can handle all crystal systems and diffraction patterns of any orientation (Rauch *et al.*, 2010 ▸; Lábár, 2022 ▸). Yet the study of metallic alloy systems, ceramics and semiconductors is still dominated by rather small unit cell phases with cubic or pseudo-cubic symmetry. Operators in TEM laboratories are therefore often tasked with the crystallographic analysis of diffraction patterns of cubic materials, so the *RAPID* program could still be of great benefit as it significantly speeds up the on-site indexing process of cubic zone axis diffraction patterns and can also be used by beginners due to its simplicity of use. As the macro code of the program is released under the GNU General Public License and is readable, it can be easily modified to meet specific needs, which may also be of interest to some users.

## 7. Availability of the macro code

The macro code of *RAPID* is published under GNU General Public License v3.0 or later at Zenodo (Weirich, 2024*c* ▸) and complies with the FAIR principles for research software (Barker *et al.*, 2022 ▸).

## Supplementary Material

Supplementary tables: log files from <it>RAPID</it> for the shown examples. DOI: 10.1107/S1600576724010215/te5143sup1.pdf
