# Supplementary material for: RAPID: an ImageJ macro for indexing electron diffraction zone axis spot patterns of cubic materials
Source: J Appl Crystallogr. 2024 Nov 26;57(Pt 6):2017–29. doi: 10.1107/S1600576724010215 (PMC11611289; doi:10.1107/S1600576724010215)
Supplement: Supplementary file 1 [file j-57-02017-sup1.pdf]

JOURNAL OF
APPLIED
CRYSTALLOGRAPHY

Volume 57 (2024)

Supporting information for article:

RAPID, an *ImageJ* macro for indexing electron diffraction zone axis spot patterns of cubic materials

Thomas E. Weirich

Table S1 RAPID log for indexing the diffraction pattern in Figure 4 (austenitic matrix)

```
* * * RAPID - RAtio method Pattern InDexing of cubic lattices (T.E. Weirich - weirich@gfe.rwth-aachen.de) * * *

Indexing is done for a cubic F-lattice
WARNING! hkl indices of the two basis vectors are user-restricted to a maximum value of 2 !
WARNING! output is user-restricted to positive [uvw] indices !
WARNING! calculated d-values and lattice parameters are based on the camera constant CC = 590 [Ang*Pixel]
WARNING! output contains only unit cells in the range between a(min) = 0  Ang. and a(max) = 20 Ang. !

* * *  INITIAL CALCULATIONS FOR JOB <SAED X5CRNIMO EXAMPLE.JPG>  * * *

Type: TEM SAED spot pattern

d-value of reflection line 1 (red) = 2.149 Ang. (from 4 reflections) // rotation angle: 24.42 degr.
d-value of reflection line 2 (green) = 1.304 Ang. (from 2 reflections)
d-value of reflection line 3 (blue) = 1.116 Ang. (from 2 reflections)

ratio 2-1 = 1.648    allowed variation:  +/- 0.033 (2 %)
angle 2-1 = 89.03    allowed variation:  +/- 2 degr.

ratio 3-1 = 1.926    allowed variation:  +/- 0.039 (2 %)
angle 3-1 = 59.25    allowed variation:  +/- 2 degr.

* * * INDEXING * * *

a , lattice type , [uvw] , hkl(1 red) , hkl(2 green) , calc. ratio 2-1 , calc angle 2-1 , hkl(3 blue) , calc ratio 3-1 , calc angle 3-1,
( R-value for ratios [%] ; max. angle deviation [degr.] )

3.704,F,[ 1 1 2 ],-1 -1 1,2 -2 0,1.633,90,-3 1 1,1.915,58.52  ---  0.7275 ; 0.97
3.704,F,[ 1 2 1 ],-1 1 -1,-2 0 2,1.633,90,1 1 -3,1.915,58.52  ---  0.7275 ; 0.97
3.704,F,[ 2 1 1 ],-1 1 1,0 -2 2,1.633,90,-1 3 -1,1.915,58.52  ---  0.7275 ; 0.97
3.704,F,[ 2 1 1 ],1 -1 -1,0 2 -2,1.633,90,1 -3 1,1.915,58.52  ---  0.7275 ; 0.97
3.704,F,[ 1 2 1 ],1 -1 1,2 0 -2,1.633,90,-1 -1 3,1.915,58.52  ---  0.7275 ; 0.97
3.704,F,[ 1 1 2 ],1 1 -1,-2 2 0,1.633,90,3 -1 -1,1.915,58.52  ---  0.7275 ; 0.97
```

Table S2 RAPID log for indexing the diffraction pattern in Figure 5 ($M_{23}C_6$ carbide)

```
* * * RAPID - RAtio method Pattern InDexing of cubic lattices (T.E. Weirich - weirich@gfe.rwth-aachen.de) * * *

Indexing is done for a cubic F-lattice
WARNING! hkl indices of the two basis vectors are user-restricted to a maximum value of 2 !
WARNING! output is user-restricted to positive [uvw] indices !
WARNING! calculated d-values and lattice parameters are based on the camera constant CC = 590 [Ang*Pixel]
WARNING! output contains only unit cells in the range between a(min) = 0  Ang. and a(max) = 20 Ang. !

* * *  INITIAL CALCULATIONS FOR JOB <SAED X5CRNIMO EXAMPLE.JPG>  * * *

Type: TEM SAED spot pattern

d-value of reflection line 1 (red) = 6.414 Ang. (from 8 reflections) // rotation angle: 4.05 degr.
d-value of reflection line 2 (green) = 3.904 Ang. (from 6 reflections)
d-value of reflection line 3 (blue) = 3.332 Ang. (from 4 reflections)

ratio 2-1 = 1.643    allowed variation:  +/- 0.033 (2 %)
angle 2-1 = 89.62    allowed variation:  +/- 2 degr.

ratio 3-1 = 1.925    allowed variation:  +/- 0.039 (2 %)
angle 3-1 = 58.48    allowed variation:  +/- 2 degr.

* * * INDEXING * * *

a , lattice type , [uvw] , hkl(1 red) , hkl(2 green) , calc. ratio 2-1 , calc angle 2-1 , hkl(3 blue) , calc ratio 3-1 , calc angle 3-1,
( R-value for ratios [%] ; max. angle deviation [degr.] )

11.069,F,[ 1 1 2 ],-1 -1 1,2 -2 0,1.633,90,-3 1 1,1.915,58.52  ---  0.5605 ; 0.38
11.069,F,[ 1 2 1 ],-1 1 -1,-2 0 2,1.633,90,1 1 -3,1.915,58.52  ---  0.5605 ; 0.38
11.069,F,[ 2 1 1 ],-1 1 1,0 -2 2,1.633,90,-1 3 -1,1.915,58.52  ---  0.5605 ; 0.38
11.069,F,[ 2 1 1 ],1 -1 -1,0 2 -2,1.633,90,1 -3 1,1.915,58.52  ---  0.5605 ; 0.38
11.069,F,[ 1 2 1 ],1 -1 1,2 0 -2,1.633,90,-1 -1 3,1.915,58.52  ---  0.5605 ; 0.38
11.069,F,[ 1 1 2 ],1 1 -1,-2 2 0,1.633,90,3 -1 -1,1.915,58.52  ---  0.5605 ; 0.38
```

Table S3 RAPID log for indexing the diffraction pattern in Figure 6 (Fe_3O_4 nanoparticle, P test)

```
* * * RAPID - RAtio method Pattern InDexing of cubic lattices (T.E. Weirich - weirich@gfe.rwth-aachen.de) * * *

Indexing is done for a cubic P-lattice
WARNING! hkl indices of the two basis vectors are user-restricted to a maximum value of 2 !
WARNING! output is user-restricted to positive [uvw] indices !
WARNING! calculated d-values and lattice parameters are based on the camera constant CC = 281 [Ang*Pixel]
WARNING! output contains only unit cells in the range between a(min) = 0  Ang. and a(max) = 20 Ang. !

* * *  INITIAL CALCULATIONS FOR JOB <FFT OF FE3O4.JPG>  * * *

Type: TEM SAED spot pattern

d-value of reflection line 1 (red) = 2.962 Ang. (from 4 reflections) // rotation angle: 146.94 degr.
d-value of reflection line 2 (green) = 2.922 Ang. (from 4 reflections)
d-value of reflection line 3 (blue) = 2.985 Ang. (from 4 reflections)

ratio 2-1 = 1.014    allowed variation:  +/- 0.02 (2 %)
angle 2-1 = 59.29    allowed variation:  +/- 2 degr.

ratio 3-1 = 0.992    allowed variation:  +/- 0.02 (2 %)
angle 3-1 = 59.98    allowed variation:  +/- 2 degr.

* * * INDEXING * * *

a , lattice type , [uvw] , hkl(1 red) , hkl(2 green) , calc. ratio 2-1 , calc angle 2-1 , hkl(3 blue) , calc ratio 3-1 , calc angle 3-1,
( R-value for ratios [%] ; max. angle deviation [degr.] )

4.181,P,[ 1 1 1 ],-1 0 1,0 -1 1,1,60,-1 1 0,1,60  ---  1.0967 ; 0.71
4.181,P,[ 1 1 1 ],-1 1 0,-1 0 1,1,60,0 1 -1,1,60  ---  1.0967 ; 0.71
4.181,P,[ 1 1 1 ],0 -1 1,1 -1 0,1,60,-1 0 1,1,60  ---  1.0967 ; 0.71
4.181,P,[ 1 1 1 ],0 1 -1,-1 1 0,1,60,1 0 -1,1,60  ---  1.0967 ; 0.71
4.181,P,[ 1 1 1 ],1 -1 0,1 0 -1,1,60,0 -1 1,1,60  ---  1.0967 ; 0.71
4.181,P,[ 1 1 1 ],1 0 -1,0 1 -1,1,60,1 -1 0,1,60  ---  1.0967 ; 0.71
```

Table S4 RAPID log for indexing the diffraction pattern in Figure 6 (Fe_3O_4 nanoparticle, I test)

```
* * * RAPID - RAtio method Pattern InDexing of cubic lattices (T.E. Weirich - weirich@gfe.rwth-aachen.de) * * *

Indexing is done for a cubic I-lattice
WARNING! hkl indices of the two basis vectors are user-restricted to a maximum value of 2 !
WARNING! output is user-restricted to positive [uvw] indices !
WARNING! calculated d-values and lattice parameters are based on the camera constant CC = 281 [Ang*Pixel]
WARNING! output contains only unit cells in the range between a(min) = 0  Ang. and a(max) = 20 Ang. !

* * *  INITIAL CALCULATIONS FOR JOB <FFT OF FE3O4.JPG>  * * *

Type: TEM SAED spot pattern

d-value of reflection line 1 (red) = 2.962 Ang. (from 4 reflections) // rotation angle: 146.94 degr.
d-value of reflection line 2 (green) = 2.922 Ang. (from 4 reflections)
d-value of reflection line 3 (blue) = 2.985 Ang. (from 4 reflections)

ratio 2-1 = 1.014    allowed variation:  +/- 0.02 (2 %)
angle 2-1 = 59.29    allowed variation:  +/- 2 degr.

ratio 3-1 = 0.992    allowed variation:  +/- 0.02 (2 %)
angle 3-1 = 59.98    allowed variation:  +/- 2 degr.

* * * INDEXING * * *

a , lattice type , [uvw] , hkl(1 red) , hkl(2 green) , calc. ratio 2-1 , calc angle 2-1 , hkl(3 blue) , calc ratio 3-1 , calc angle 3-1,
( R-value for ratios [%] ; max. angle deviation [degr.] )

4.181,I,[ 1 1 1 ],-1 0 1,0 -1 1,1,60,-1 1 0,1,60  ---  1.0967 ; 0.71
4.181,I,[ 1 1 1 ],-1 1 0,-1 0 1,1,60,0 1 -1,1,60  ---  1.0967 ; 0.71
4.181,I,[ 1 1 1 ],0 -1 1,1 -1 0,1,60,-1 0 1,1,60  ---  1.0967 ; 0.71
4.181,I,[ 1 1 1 ],0 1 -1,-1 1 0,1,60,1 0 -1,1,60  ---  1.0967 ; 0.71
4.181,I,[ 1 1 1 ],1 -1 0,1 0 -1,1,60,0 -1 1,1,60  ---  1.0967 ; 0.71
4.181,I,[ 1 1 1 ],1 0 -1,0 1 -1,1,60,1 -1 0,1,60  ---  1.0967 ; 0.71
```

Table S5 RAPID log for indexing the diffraction pattern in Figure 6 (Fe_3O_4 nanoparticle, F test)

```
* * * RAPID - RAtio method Pattern InDexing of cubic lattices (T.E. Weirich - weirich@gfe.rwth-aachen.de) * * *

Indexing is done for a cubic F-lattice
WARNING! hkl indices of the two basis vectors are user-restricted to a maximum value of 2 !
WARNING! output is user-restricted to positive [uvw] indices !
WARNING! calculated d-values and lattice parameters are based on the camera constant CC = 281 [Ang*Pixel]
WARNING! output contains only unit cells in the range between a(min) = 0  Ang. and a(max) = 20 Ang. !

* * *  INITIAL CALCULATIONS FOR JOB <FFT OF FE3O4.JPG>  * * *

Type: TEM SAED spot pattern

d-value of reflection line 1 (red) = 2.962 Ang. (from 4 reflections) // rotation angle: 146.94 degr.
d-value of reflection line 2 (green) = 2.922 Ang. (from 4 reflections)
d-value of reflection line 3 (blue) = 2.985 Ang. (from 4 reflections)

ratio 2-1 = 1.014    allowed variation:  +/- 0.02 (2 %)
angle 2-1 = 59.29    allowed variation:  +/- 2 degr.

ratio 3-1 = 0.992    allowed variation:  +/- 0.02 (2 %)
angle 3-1 = 59.98    allowed variation:  +/- 2 degr.

* * * INDEXING * * *

a , lattice type , [uvw] , hkl(1 red) , hkl(2 green) , calc. ratio 2-1 , calc angle 2-1 , hkl(3 blue) , calc ratio 3-1 , calc angle 3-1,
( R-value for ratios [%] ; max. angle deviation [degr.] )

8.363,F,[ 1 1 1 ],-2 0 2,0 -2 2,1,60,-2 2 0,1,60  ---  1.0967 ; 0.71
8.363,F,[ 1 1 1 ],-2 2 0,-2 0 2,1,60,0 2 -2,1,60  ---  1.0967 ; 0.71
8.363,F,[ 1 1 1 ],0 -2 2,2 -2 0,1,60,-2 0 2,1,60  ---  1.0967 ; 0.71
8.363,F,[ 1 1 1 ],0 2 -2,-2 2 0,1,60,2 0 -2,1,60  ---  1.0967 ; 0.71
8.363,F,[ 1 1 1 ],2 -2 0,2 0 -2,1,60,0 -2 2,1,60  ---  1.0967 ; 0.71
8.363,F,[ 1 1 1 ],2 0 -2,0 2 -2,1,60,2 -2 0,1,60  ---  1.0967 ; 0.71
```

Table S6 RAPID log for indexing the diffraction pattern in Figure 7 (alloy AlZn5Mg, P-test)

```
* * * RAPID - RAtio method Pattern InDexing of cubic lattices (T.E. Weirich - weirich@gfe.rwth-aachen.de) * * *

Indexing is done for a cubic P-lattice
WARNING! hkl indices of the two basis vectors are user-restricted to a maximum value of 3 !
WARNING! output is user-restricted to positive [uvw] indices !
WARNING! output contains only results with sum N1 + N2 in the range between N(min) = 0 and N(max) = 20 !
WARNING! Kikuchi patterns may require a significant increase in the range of error for ratios and angles !

* * *  INITIAL CALCULATIONS FOR JOB <ALLOY ALZN5MG KIKUCHI BANDS.JPG>  * * *

Type: TEM Kikuchi pattern

Length of line 1 (red) = 195.7 pixel (from band width) // rotation angle: 40.86 degr.
Length of line 2 (green) = 227.5 pixel (from band width)
Length of line 3 (blue) = 230.1 pixel (from band width)

ratio 2-1 = 1.163    allowed variation:  +/- 0.023 (2 %)
angle 2-1 = 63.91    allowed variation:  +/- 2 degr.

ratio 3-1 = 1.176    allowed variation:  +/- 0.024 (2 %)
angle 3-1 = 64.97    allowed variation:  +/- 2 degr.

* * * INDEXING * * *

N , lattice type , [uvw] , hkl(1 red) , hkl(2 green) , calc. ratio 2-1 , calc angle 2-1 , hkl(3 blue) , calc ratio 3-1 , calc angle 3-1,
( R-value for ratios [%] ; max. angle deviation [degr.] )

10,P,[ 3 5 9 ],-3 0 1,-1 -3 2,1.183,65,-2 3 -1,1.183,65  ---  1.1543 ; 1.09
10,P,[ 3 9 5 ],-3 1 0,-2 -1 3,1.183,65,-1 2 -3,1.183,65  ---  1.1543 ; 1.09
10,P,[ 9 5 3 ],-1 0 3,1 -3 2,1.183,65,-2 3 1,1.183,65  ---  1.1543 ; 1.09
10,P,[ 9 3 5 ],-1 3 0,-2 1 3,1.183,65,1 2 -3,1.183,65  ---  1.1543 ; 1.09
10,P,[ 5 3 9 ],0 -3 1,3 -2 -1,1.183,65,-3 -1 2,1.183,65  ---  1.1543 ; 1.09
10,P,[ 5 9 3 ],0 -1 3,3 -2 1,1.183,65,-3 1 2,1.183,65  ---  1.1543 ; 1.09
10,P,[ 5 9 3 ],0 1 -3,-3 2 -1,1.183,65,3 -1 -2,1.183,65  ---  1.1543 ; 1.09
10,P,[ 5 3 9 ],0 3 -1,-3 2 1,1.183,65,3 1 -2,1.183,65  ---  1.1543 ; 1.09
10,P,[ 9 3 5 ],1 -3 0,2 -1 -3,1.183,65,-1 -2 3,1.183,65  ---  1.1543 ; 1.09
10,P,[ 9 5 3 ],1 0 -3,-1 3 -2,1.183,65,2 -3 -1,1.183,65  ---  1.1543 ; 1.09
10,P,[ 3 9 5 ],3 -1 0,2 1 -3,1.183,65,1 -2 3,1.183,65  ---  1.1543 ; 1.09
10,P,[ 3 5 9 ],3 0 -1,1 3 -2,1.183,65,2 -3 1,1.183,65  ---  1.1543 ; 1.09
```

Table S7 RAPID log for indexing the diffraction pattern in Figure 7 (alloy AlZn5Mg, I-test)

```
* * * RAPID - RAtio method Pattern InDexing of cubic lattices (T.E. Weirich - weirich@gfe.rwth-aachen.de) * * *

Indexing is done for a cubic I-lattice
WARNING! hkl indices of the two basis vectors are user-restricted to a maximum value of 3 !
WARNING! output is user-restricted to positive [uvw] indices !
WARNING! output contains only results with sum N1 + N2 in the range between N(min) = 0 and N(max) = 20 !
WARNING! Kikuchi patterns may require a significant increase in the range of error for ratios and angles !

* * *  INITIAL CALCULATIONS FOR JOB <ALLOY ALZN5MG KIKUCHI BANDS.JPG>  * * *

Type: TEM Kikuchi pattern

Length of line 1 (red) = 195.7 pixel (from band width) // rotation angle: 40.86 degr.
Length of line 2 (green) = 227.5 pixel (from band width)
Length of line 3 (blue) = 230.1 pixel (from band width)

ratio 2-1 = 1.163    allowed variation:  +/- 0.023 (2 %)
angle 2-1 = 63.91    allowed variation:  +/- 2 degr.

ratio 3-1 = 1.176    allowed variation:  +/- 0.024 (2 %)
angle 3-1 = 64.97    allowed variation:  +/- 2 degr.

* * * INDEXING * * *

N , lattice type , [uvw] , hkl(1 red) , hkl(2 green) , calc. ratio 2-1 , calc angle 2-1 , hkl(3 blue) , calc ratio 3-1 , calc angle 3-1,
( R-value for ratios [%] ; max. angle deviation [degr.] )

10,I,[ 3 5 9 ],-3 0 1,-1 -3 2,1.183,65,-2 3 -1,1.183,65  ---  1.1543 ; 1.09
10,I,[ 3 9 5 ],-3 1 0,-2 -1 3,1.183,65,-1 2 -3,1.183,65  ---  1.1543 ; 1.09
10,I,[ 9 5 3 ],-1 0 3,1 -3 2,1.183,65,-2 3 1,1.183,65  ---  1.1543 ; 1.09
10,I,[ 9 3 5 ],-1 3 0,-2 1 3,1.183,65,1 2 -3,1.183,65  ---  1.1543 ; 1.09
10,I,[ 5 3 9 ],0 -3 1,3 -2 -1,1.183,65,-3 -1 2,1.183,65  ---  1.1543 ; 1.09
10,I,[ 5 9 3 ],0 -1 3,3 -2 1,1.183,65,-3 1 2,1.183,65  ---  1.1543 ; 1.09
10,I,[ 5 9 3 ],0 1 -3,-3 2 -1,1.183,65,3 -1 -2,1.183,65  ---  1.1543 ; 1.09
10,I,[ 5 3 9 ],0 3 -1,-3 2 1,1.183,65,3 1 -2,1.183,65  ---  1.1543 ; 1.09
10,I,[ 9 3 5 ],1 -3 0,2 -1 -3,1.183,65,-1 -2 3,1.183,65  ---  1.1543 ; 1.09
10,I,[ 9 5 3 ],1 0 -3,-1 3 -2,1.183,65,2 -3 -1,1.183,65  ---  1.1543 ; 1.09
10,I,[ 3 9 5 ],3 -1 0,2 1 -3,1.183,65,1 -2 3,1.183,65  ---  1.1543 ; 1.09
10,I,[ 3 5 9 ],3 0 -1,1 3 -2,1.183,65,2 -3 1,1.183,65  ---  1.1543 ; 1.09
```

Table S8 RAPID log for indexing the diffraction pattern in Figure 7 (alloy AlZn5Mg, F-test)

```
* * * RAPID - RAtio method Pattern InDexing of cubic lattices (T.E. Weirich - weirich@gfe.rwth-aachen.de) * * *

Indexing is done for a cubic F-lattice
WARNING! hkl indices of the two basis vectors are user-restricted to a maximum value of 3 !
WARNING! output is user-restricted to positive [uvw] indices !
WARNING! output contains only results with sum N1 + N2 in the range between N(min) = 0 and N(max) = 20 !
WARNING! Kikuchi patterns may require a significant increase in the range of error for ratios and angles !

* * *  INITIAL CALCULATIONS FOR JOB <ALLOY ALZN5MG KIKUCHI BANDS.JPG>  * * *

Type: TEM Kikuchi pattern

Length of line 1 (red) = 195.7 pixel (from band width) // rotation angle: 40.86 degr.
Length of line 2 (green) = 227.5 pixel (from band width)
Length of line 3 (blue) = 230.1 pixel (from band width)

ratio 2-1 = 1.163    allowed variation:  +/- 0.023 (2 %)
angle 2-1 = 63.91    allowed variation:  +/- 2 degr.

ratio 3-1 = 1.176    allowed variation:  +/- 0.024 (2 %)
angle 3-1 = 64.97    allowed variation:  +/- 2 degr.

* * * INDEXING * * *

N , lattice type , [uvw] , hkl(1 red) , hkl(2 green) , calc. ratio 2-1 , calc angle 2-1 , hkl(3 blue) , calc ratio 3-1 , calc angle 3-1,
( R-value for ratios [%] ; max. angle deviation [degr.] )

9,F,[ 1 4 1 ],-2 0 2,1 -1 3,1.173,64.76,-3 1 -1,1.173,64.76  ---  0.5558 ; 0.85
9,F,[ 1 1 4 ],-2 2 0,-3 -1 1,1.173,64.76,1 3 -1,1.173,64.76  ---  0.5558 ; 0.85
9,F,[ 4 1 1 ],0 -2 2,1 -3 -1,1.173,64.76,-1 1 3,1.173,64.76  ---  0.5558 ; 0.85
9,F,[ 4 1 1 ],0 2 -2,-1 3 1,1.173,64.76,1 -1 -3,1.173,64.76  ---  0.5558 ; 0.85
9,F,[ 1 1 4 ],2 -2 0,3 1 -1,1.173,64.76,-1 -3 1,1.173,64.76  ---  0.5558 ; 0.85
9,F,[ 1 4 1 ],2 0 -2,-1 1 -3,1.173,64.76,3 -1 1,1.173,64.76  ---  0.5558 ; 0.85
```
